# The interaction of shock waves with biological tissue – momentum transfer, the key for tissue stimulation and fragmentation

**DOI:** 10.1097/JS9.0000000000002261

**Published:** 2025-02-27

**Authors:** Othmar Wess, Juergen Mayer

**Affiliations:** Department Applied Research, Storz Medical AG, Taegerwilen, Thurgau, Switzerland

**Keywords:** fragmentation, momentum transfer, neurostimulation, shock waves, shock wave therapy

## Abstract

**Background:**

Shock waves in medicine have gained enormous importance and have spread since 1980, and the first kidney stone was successfully fragmented in a patient in Munich. Meanwhile, the spectrum of medical applications of shock waves ranges from powerful fragmentation of kidney stones to diverse indications such as wound healing, chronic pelvic pain, spasticity, erectile dysfunction, and others, to neuro-stimulation in the context of Alzheimer’s disease. A comprehensive working mechanism for this diverse field of medical indications is still missing.

**Objective:**

Investigation of the physical basis of the working mechanism of shock waves in medical applications.

**Methods:**

We developed a model based on the mechanical forces generated by the momentum transfer at the acoustic interfaces of different layers of biological tissue. The generated forces are strong enough to crash brittle material and provide an adequate mechanical stimulus to activate **mechano-transduction** and **mechano-sensory-transduction** with nerve stimulation, thereby affecting the neural memory function of the central nervous system.

**Results:**

The key to generating appropriate forces in the millisecond range is the mechanism of momentum transfer at the interfaces between tissue layers with different acoustic impedances. According to Newton’s laws of motion, a change in momentum (momentum transfer) generates force *F* = d*P*/d*t*. The inherent shear forces can stretch biological membranes to release biomolecules such as vascular endothelial growth factor and nitric oxide. A most favorable feature of this mechanism is the selective effect on soft tissue interfaces and small tissue inhomogeneities to generate small forces in the range of few (≤10) Newton to stimulate tissue and nerve cells, while the same shock wave can generate forces ≥200 Newton and more on hard tissue interfaces such as bones or stones.

**Conclusion:**

The mechanism of momentum transfer is the basis for mechano-transduction and mechano-sensory transduction. It offers the opportunity to stimulate peripheral nerves and modify the motor reflex patterns of “pathologic” reflexes by hyper stimulation. The new technique of transcranial pulse stimulation may be based on direct stimulation and reactivation of neurons in the brain. Momentum transfer is the basic physical mechanism and the initiator for successive biological processes in medical shock wave therapy.

## Introduction

Shock waves have been in use since 1980^[1]^. First, kidney stones were fragmented by focusing extracorporeally generated waves through the intact skin onto stones within the kidney^[2-5]^. At the beginning of the shock wave therapy era, fragmentation of brittle body stones, such as kidney and gallbladder stones, was the primary goal of shock wave application. To date, all types of brittle stone materials^[6-10]^ have been suitable for shock wave fragmentation. Remarkably, the mechanism of stone fragmentation remains under discussion^[11]^.

**HIGHLIGHTS**

- Interaction of shock waves with biological tissue.
- The momentum turned out to be a significant feature of shock waves.
- Shock waves stimulate mechano-transduction processes in biological tissues.
- Shock waves stimulate peripheral and central nerves.
- Neurostimulation TPS, hope for Alzheimer patients.

Some years after the first human application of stone fragmentation, shock waves have been shown to stimulate various soft and hard tissues such as skin, muscle, and bones. We learned that shock waves are strong enough to break brittle materials such as human calculi and, simultaneously, gentle enough to stimulate soft tissue without causing tissue lesions. To date, an increasing number of medical applications in different fields of urology^[12-15]^, orthopedics^[16-19]^, cardiology^[20-23]^, neurology^[24-28]^, and chronic pain^[12,13]^ been subject to shock wave therapy. This extraordinarily wide spectrum, from powerful fragmentation of brittle stones to soft stimulation of Alzheimer’s patients, is not only based on the power level of applied shock waves but also on the type of soft and hard tissue. Shock waves act selectively on various tissues.

In 1997, a working group published a list of parameters that characterize the features of shock waves^[5]^. The goal was to find correlations between medical effectiveness and shock wave features. The most important parameters, such as peak pressure, energy, rise time, and focal size, have not yet been clearly answered.

In case of “non-destructive” applications in the wide field of stimulation of various soft and hard tissues the parameter “energy-flux-density” (ED) is widely used to characterize the power of the applied shock waves. A correlation between this parameter and medical success has not yet been established, although it provides a certain impression of the intensity of the treatment.

The question of why and how shock waves possess the extremely advantageous feature of high destruction power on stones without causing significant tissue lesions and simultaneously offering soft stimulation of numerous kinds of tissues remains open.

This study attempts to elucidate the working mechanism of shock waves in medicine in terms of their physical aspects.

For the first time in four decades of medical shock wave history we can provide answers on the above mentioned questions by the theory of momentum transfer. We can provide the previously unrecognized physical basis for millions of successful medical treatments with current shock wave devices and raise the level of understanding the physics of shock wave therapy.

Because shock waves are mechanical waves, we cannot expect features other than the intrinsic characteristics of a mechanical object such as pressure, tensile forces, and other Newtonian quantities. Shock waves affect the healing mechanism of various medical diseases by simply applying the same type of mechanical force in different configurations and power levels.

We developed a theory on how shock waves generate adequate forces in several biological tissues. “Adequate” means that the duration of the generated forces match with the biological response times in the range of milliseconds.

Many physiological processes occur within milliseconds (ms). For example, the typical duration of an action potential of nerve cells is approx. 1 ms and is far slower than the oscillation period of ultrasound. The discrepancy between the time frame of physiological processes and the oscillation period of ultrasound waves may be the reason why continuous ultrasound is less suitable for the stimulation of healing processes, even when applied in a pulsed manner.

**Figure 1.**
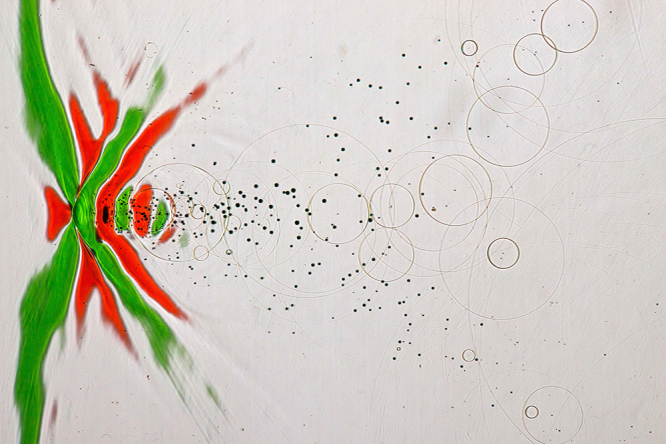
Shock wave pulse visualized by color-schlieren-optic. Shock waves are pressure waves traveling, e.g., through water as pressure distortions with a velocity of approx. 1500 m/s. Usually, the pressure distortions are not visible by the naked eye. A color optical schlieren apparatus^[29]^ can visualize gradients of the pressure distortions, which are here displayed in red (pressure rise) and in green (pressure decline). The shock wave is traveling from right to left. The first small red triangle on the symmetry axis left depicts the area of rapid pressure rise. From the peak, the pressure decreases, displayed in green color until the minimum pressure is reached and is again raising (displayed in red). Several smaller reverberations with alternating pressures and according to pressure gradients are displayed further to the right. After the first minimum pressure (right rim of the first green line) a field of cavitation bubbles occurs. Some of the bubbles collapse already and radiate secondary spherical shock waves depicted by the circles at the right side of the frame. Shock waves like the one displayed are transmitted from outside the body through the intact skin into deeper target areas of the tissue, where they stimulate tissue interfaces or fragment body stones via the mechanism of momentum transfer.

Shock waves are characterized by solitary pressure pulses with a duration of approx. 1 microsecond (1 µs) which corresponds to the wavelength of the approx. 1.5 mm in soft tissue. This relatively short pulse length is an important feature because it enables the focusing of shock waves to small confined treatment volumes of several mm^3^. Despite a short pulse duration (approx. 1 µs) shock waves are audible, and you can feel them, meaning that mechanical stimulation of physiological functions is possible (see Fig. 1).

Beyond these qualities, shock waves have the additional capability to interact with living tissues and stimulate physiological healing mechanisms.

Mechanotransduction is considered to be responsible for acting on cell membranes by stretching, pressing, or deforming membranes. Mechanical forces open ion channels and cell pores, enabling ions and bio-chemicals to pass through the cellular membrane. The time required for this mechanism is in the millisecond (kHz) range. In comparison, ultrasound in the range of microseconds (MHz) is too fast to stimulate mechanotransduction. The highest frequency detected by the highly sensitive hearing cells in the human ear did not exceed 20 kHz.

The generation of adequate ms forces (kHz) at the selected tissue and how shock waves provide mechanical stimuli suitable for mechano-transduction and neuro-stimulation will be described.

## Materials and methods

First, we investigated the mechanism of generation of the appropriate forces by shock waves and their mechanical effects on stones and soft and hard tissues on the basis of experimental data available in Ref.^[29]^ and “additional material” of this paper. Second, we developed a memory-based theory of shock wave treatment for certain chronic diseases, such as chronic pain and healing of chronic wounds, based on the hyper-stimulation of nerve cells in a neural memory network. Third, we hypothesized that shock waves may act on the central nervous system (CNS) in cases of transcranial pulse stimulation (TPS) for Alzheimer`s disease.

## Results

### How shock waves generate forces in in hard and soft biological tissue

To understand the mechanism of the generation of mechanical forces in biological tissue, we must examine the characteristics of shock waves and their interactions with different types of biological tissue.

#### Characteristics of shockwaves

Shock waves are characterized by several parameters such as:

- high peak pressure,
- steep pressure rise,
- focal dimensions,
- energy content,
- momentum.

Of the listed parameters, energy-based parameters such as energy flux density (EFD), measured in millijoules per millimeter squared (mJ/mm^2^), are used to characterize the power of a shock wave. This parameter may provide an impression of the strength of a shock wave, but the energy per se is not the reason for this effect. Energy requires a mechanism (motor) to generate forces and momentum, which in turn may affect biological tissues and initiate the desired healing process

The **momentum** of a shock wave is one of the most relevant features, because it is the basis for the mechanical stimulation of soft tissue and fragmentation of brittle materials such as kidney stones and other solid materials. The importance of momentum in shockwave applications in medicine has been overlooked and unnoticed for many years.

Momentum is a prerequisite for momentum transfer. To date, this essential parameter has rarely been considered in the corresponding literature and has not yet been specified in the IEC Standard 61846. Wess and Mayer (2018)^[29]^ measured the momentum of a shock by momentum transfer on artificial kidney stones. Details of the measurement are described in Ref.^[29]^ and in “Supplemental files” of this paper (available at: http://links.lww.com/JS9/D976).

The momentum of a shock wave is defined as follows: (momentum is a vector featuring number and direction. For simplicity, we restrict ourselves to the special case of linear motions, and perpendicular reflection only reverses the direction and sign of the momentum.)

Within a cross section area *A* the momentum *P* is given by:

$$P=A\int_{0}^{t}p(t)dt,$$

or momentum density:

$$P/A\int_{0}^{t}p(t)dt,$$

with *P* = pressure, *A* = cross section area, *t* = time.

**Figure 2.**
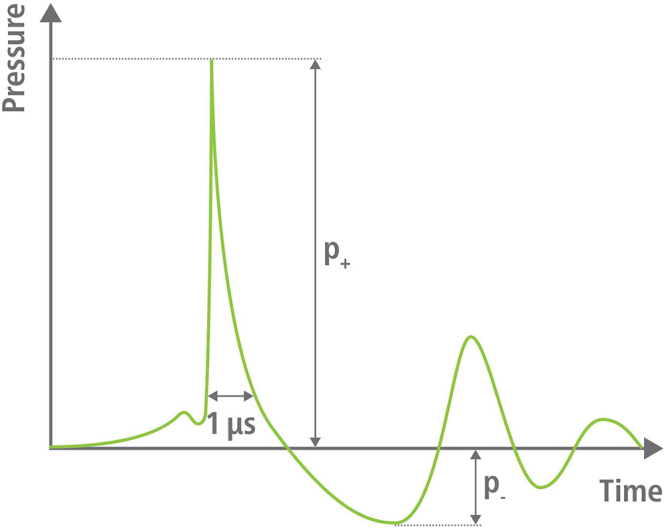
Typical pressure values measured on the axis of the shock wave propagation. The shock wave travels from left to right as in Fig. 1, starts with a rapid rise of a few nanoseconds and declines in approx. 1 µs to 0, followed by tensile pressure of several microseconds. The tail displays decaying alternating pressure which appears in Fig. 1 in red in green colors.

**Figure 3.**
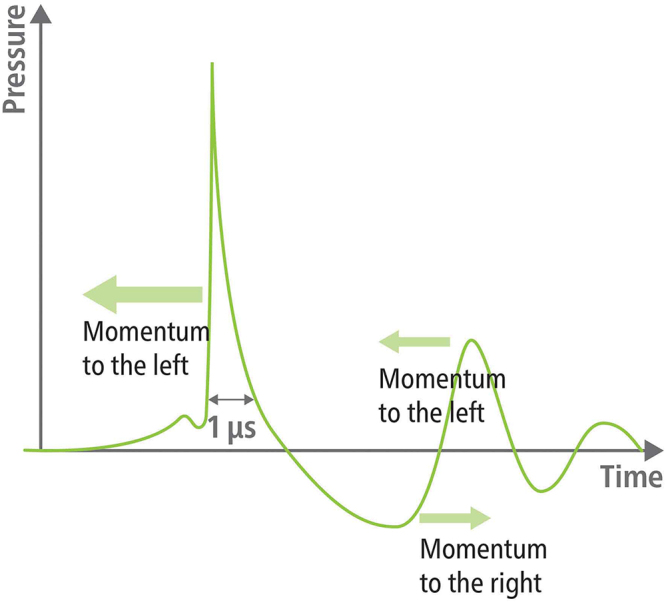
According to the positive part of the pressure curve a momentum in the propagation direction of the shock wave (to the left) is generated followed, due to tensile pressures, by momentum to the opposite direction (to the right). Further decaying reverberations follow with alternating directions.

Owing to the positive pressure values in Fig. 2, the momentum is in line with the propagation direction of the shock wave, whereas the negative pressure part features momentum in the opposite direction, as shown in Fig. 3.

### Reflection of shock waves at acoustic interfaces

At the interfaces between different organs and inter-organ structures, a part of the impinging shock wave is transmitted into the adjacent area, and another part is reflected. The reflecting structures can be visualized by the common technique of “echo sonography” which makes use of the reflected ultrasound pulses to generate 2- or 3-dimensional images of acoustic properties of the tissue.

**Figure 4.**
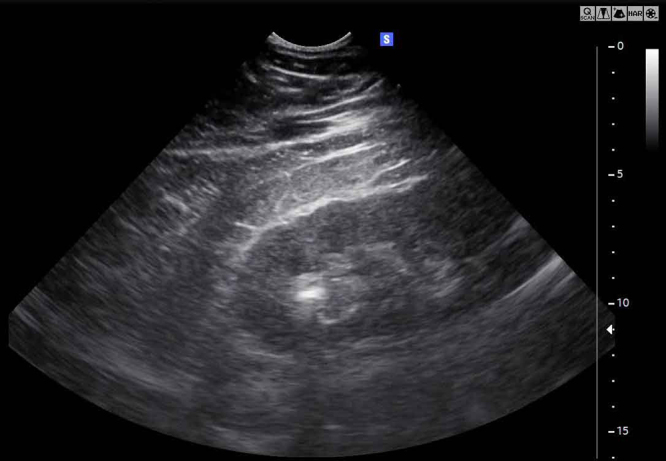
Interfaces of various tissue layers in front of a kidney with a stone are depicted in an ultrasound echo image. Ultrasound and shock waves both are acoustic waves and are partly transmitted and partly reflected according to the reflection coefficient *R* for the present impedance difference. For the tissue layers in front of the kidney, transmission of the ultrasound waves dominates due to similar *Z*-values (*Z*_2_ ≈ *Z*_1_), for the stone, reflection of ultrasound reflection dominates due to significantly different *Z*-values (*Z*_2_ ≠ *Z*_1_). The lower part of the image shows a bright stone reflex, which is followed by a dark shadow, indicating that almost no ultrasound had passed the stone.

An example of a sonographic image of a kidney with caliceal stones and overlying tissue layers is shown in Fig. 4.

Shock waves propagating in media, such as the human body with soft and hard tissues, are attenuated and reflected at interfaces with different acoustic impedances. The acoustic impedance *Z* = *ρc* (kg/m^2^s) of a medium is characterized by the product of the density *ρ* (kg/m^3^) and propagation velocity *c* (m/s) (see Table 1).

**Table 1:** Some selected impedance values of some tissues and stones

Impedance	*Z = ρc* (kg/m^2^s)
Air	410
Fat	1.34 × 10^6^
Water	1.49 × 10^6^
Muscle	1.63 × 10^6^
Brain	1.56 × 10^6^
Bone	6.12 × 10^6^
Stone	6-14 × 10^6^

### Reflection coefficient R

The amplitude of the shock waves traveling from medium 1 with impedance Z_1_ to medium 2 with impedance Z_2_ depends on the reflection coefficient, which is given by:

*R* = (*Z*_2_ − *Z*_1_)/(*Z*_2_ + *Z*_1_),

*R* can vary between −1 and 1.

For *Z*_2_ = *Z*_1_ (*R* = 0) (total transmission of shock waves)

For *Z*_2_ ǂ *Z*_1_ (0 > *R* < 1) (partly transmitted, partly reflected).

For the reflection at soft/soft tissues (fat, muscle, kidney, etc.), *R* is in the range of *R* <1%; for reflection at soft/hard tissues (bone, stones, etc.), *R* is in the range of *R* ≈50–70%.

### Forces generated by shock waves − momentum transfer

The reflection of shock waves at acoustic interfaces is the basic mechanism for generating forces for medical applications.

According to the pressure slope, we have positive momentum directed along the propagation line of the shockwave when the pressure values are positive, and momentum opposite to the propagation line of the shock wave when the pressure becomes negative, as depicted in Fig. 2.

The momentum of a shock wave changes due reflection at an acoustic interface.

When shock waves are reflected at acoustic interfaces, the propagation direction changes along the direction of the momentum.

Following Newton’s laws of motion, every change in momentum ∆*P* is inherently related to a force given by *F* = ∆*P*/∆*t* or (differential *F* = d*P*/d*t*). These forces are generated by the reflection of shock waves at the borders of organs and tissue inhomogeneities within single organs because every change or reflection causes a change in momentum.

The amount of change in momentum depends on the ratio of the reflected and incident pressures at the boundary of the two different acoustic impedances.

**Figure 5.**
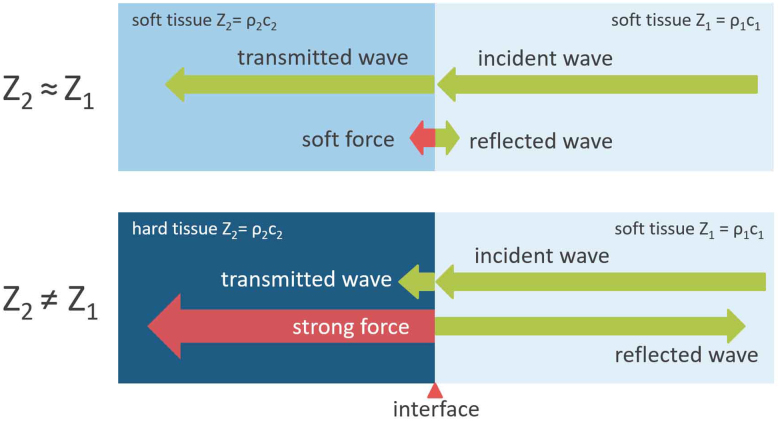
Schematic of reflection of shock waves at different acoustic interfaces. At interfaces with slight difference (*Z*_2_ ≈ *Z*_1_; e.g., between muscle tissue and fat tissue), the reflection coefficient *R* is small and the change of the momentum minor. According to Newton`s Laws of Motion the change of a momentum per time equals the strength of force generated by the change. At interfaces with significant difference (*Z*_2_ ≠ *Z*_1_; e.g., kidney parenchyma and kidney stones) the reflection coefficient *R* is closer to 1 and the change of the momentum major and the according force stronger.

It is defined by the reflectance factor R. For interfaces between soft tissue areas (*Z*_2_ ≈ *Z*_1_), the forces are minor, whereas for interfaces between soft and hard areas (*Z*_2_ ≠ *Z*_1_), they are major (see Fig. 5).

Most shock wave devices generate shock waves in a water compartment and couple the waves via a coupling device (open water bath or a closed coupling cushion with a smooth coupling membrane) into the human body tissue. The waves travel through the tissue to the target area, which may be the skin surface, underlying structures, or deeper organs, as well as the kidney stones. The shock waves pass through different tissue borders and separate areas with different acoustic features.

Because shock waves are acoustic waves, they follow the same physical rules as ultrasound waves. The reflection process of shock waves at acoustic interfaces transforms the momentum of the impinging wave into the momentum of the transmitted and reflected waves.

According to Newton’s laws of motion, the preservation of momentum requires that the sum of the reflected momentum *P*_r_ and the transmitted *P*_t_ momentum equals the original momentum *P*_o_ of the impinging wave.

*P*_o_ = *P*_t_ + *P*_r_

With reflection coefficient *R* the reflected momentum *P*_r_ is:

*P*_r_ = −*RP*_o_ (reverse propagation direction)

and transmitted momentum *P*_t_:

*P*_t_ = *P*_o_ − *P*_r_

*P*_t_ = *P*_o_ − *RP*_o_

The change ∆*P* of the momentum by reflection at an acoustic interface with the reflection coefficient *R* is given by:

∆*P* = *P*_o_ − *P*_t_ + *P*_r_

∆*P* = *P*_o_ − (*P*_o_ − *RP*_o_) + *RP*_o_

∆*P* = 2*RP*_o_

The change of the momentum depends on the reflection coefficient *R*

Examples:

1. The total reflexion (*R* = 1): *P*_r_ = − *P*_o_, *P*_t_ = 0.

∆*P* = *P*_o_ − *P*_t_ + *P*_r_

∆*P* = *P*_o_ − 0 + *P*_o_

∆*P* = 2*P*_o_

Total transmission (*R* = 0): *P*_r_ = 0, *P*_t_ = *P*_o_

∆*P* = *P*_o_ − *P*_o_ + 0

∆*P* = 0

50% reflexion (*R* = 1/2): *P*_r_ = −½*P*_o_, *P*_t_ = ½*P*_o_

∆*P* = *P*_o_ −½*P*_o_ +½*P*_o_ = *P*_o_

**∆***P* = *P*_o_

The change in momentum d*P* is inherently associated with force *F*, depending on the time required for change d*t*, and is given by:

*F* = d/d*t*

or:

*F* = ∆*P*/∆*t* (with ∆*P* = 2*RP*_o_)

Force *F* = 2*RP*_o_/∆*t*

The forces are proportional to twice the reflection coefficient (2R) and reciprocal to ∆t, which is the reflection time. For the shock wave**s** this time is approx. 1 µs (see Fig. 2).

This means that the shock wave forces generated in biological tissue depend on the momentum *P*_o_ of the impinging wave and, most importantly, on the reflection coefficient *R* of the affected tissue structures. If there are no, or minor reflecting interfaces, the high energy of a shock wave can pass through the tissue without significantly affecting the tissue.

The reflection coefficient *R* between different tissue layers is the decisive parameter determining the strength of shock waves effects in the tissue.

Some examples: A typical shock wave generator may have at a selected power level a momentum *P*_o_ = 350 µNs in a circular focal area of 5 mm diameter.

The forces generated in this area depend on the reflection coefficient *R* (see Table 2).

**Table 2:** Quantitative examples for generated forces at selected tissue interfaces

Interface	Reflection coefficient R	Generated force: *F* = 2*RP*_0_/∆*t* (*N*), *P*_o_ = 350 µNs, ∆*t* = 1 µs
Water/water (no interface)	0	0
Fat/water	0.053	37.2
Muscle/fat	0.10	70
Muscle/bone	0.58	406
Water/stone	0.78	546

The generated forces significantly differ with type of tissue (<10). The high values (>100 N) have an extremely short action time of approx. 1 µs, enough to transfer a momentum to the surrounding tissue.

### Momentum transfer, mechanism of shock waves effects on tissue

The above-developed concept of momentum transfer provides tools to understand the effects of shock waves on living tissues and their healing capabilities.

Momentum transfer generates forces at acoustic interfaces, scaled by the difference in acoustic impedances (impedance mismatch). They are weak in soft tissue and significant at interfaces with stones, bones, and lung tissue. These forces act on the affected tissue and provide momentum (*P* = *mv*) to the involved mass, meaning that the mass moves until it is attenuated by adjacent tissue.

The shock wave acts on the acoustic interface within a few microseconds. This is too fast for the direct generation of physiological processes, such as the stimulation of nerve cells.

The mechanism of momentum transfer, however, transmits momentum in microseconds to the surrounding material, such as soft tissue or hard stones and bones, and accelerates the involved material to move in a range of several cm/s. (see the calculation example in “additional material”^[29]^). This means that the involved tissue gained momentum P = mv. Because the affected mass is not free-floating but connected to adherent tissue, retarding tensile and shear forces stretches and squeezes the tissue and embedded cell membranes for several milliseconds. This pulse characteristic is well-fitted to stimulate nerve cells and open pores in cell membranes, which is considered the mechanism of mechanosensory transduction in shock wave therapeutic applications.

The mechanism of transforming a microsecond pulse into a millisecond pulse depends on the momentum of the shock wave. Ultrasound with megahertz pressure fluctuations for example, does not feature a significant momentum comparable to that of shock waves. Therefore, shock waves are ideally suited to generating stimulating forces in biological tissues, whereas ultrasound lacks significant momentum.

#### Mechano-transduction

The direct bio-chemical effect of shock waves can be excluded because shock waves are mechanical waves and only develop mechanical features, such as pressure and tensile forces.

These forces are provided by the mechanism of momentum transfer with a duration of milliseconds (ms), which is an adequate time required for physiological processes. The original shock wave stimulus acts in microseconds (µs) and is too fast for a biological response. However, the force of momentum transfer accelerates the affected mass (tissue) to inertial movement in the range of ms.

Mechano-transduction^[30,31]^ is considered to be the basic mechanism of shock wave interaction with living tissues. Mechanical forces are required to press, stretch, and shear cell membranes to open ion channels and to express substances such as eNOS, BMP, VEGF, and substance P. These bio-substances are thought to increase vasodilatation, metabolism, and initiate healing processes and are the reason for successful shock wave therapy.

#### Mechano-sensory-transduction

A mechanical stimulus of milliseconds has the ideal time duration not only to express bio-substances but also to stimulate nerve cells to generate action potentials. They travel across nerve fibers to the synaptic junctions of adjacent nerve cells in the CNS. This mechanism is known as the mechanosensory transduction^[32]^. The application of shock waves to living tissues usually implicates the stimulation of nerve cells and affects not only local processes but also neural processes in the brain.

This feature opens a wide range of treatment options for numerous diseases with a neural background.

### Shock waves and their interaction with the central nervous system (CNS) − a neural hypothesis

Many diseases, such as chronic pain in general and chronic pelvic pain, heel spurs, tennis elbow, and shoulder pain, are not fully understood without considering sensori-motor reflexes and neural memory functions. A possible mechanism has been published by Wess.^[33]^

**Figure 6.**
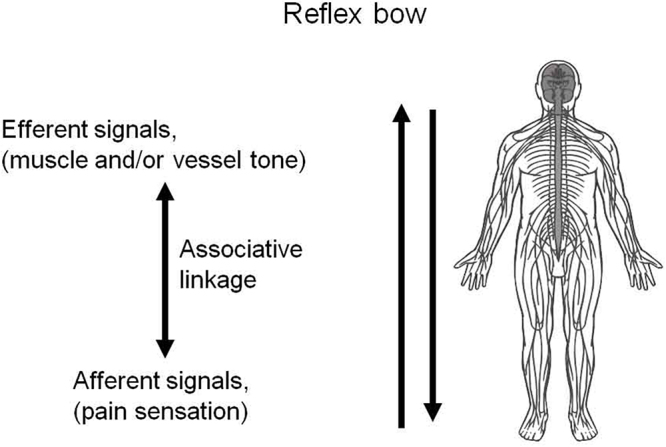
Schematic view of afferent and efferent pathways.

The theory of chronic pain and pain relief by extracorporeal shock wave treatment is briefly outlined as follows. Chronic pain without an underlying anatomical disorder is considered a pathological control function of memory. Conditioned reflexes are engraved memory traces that link the sensory input of afferent signals with the motor response of efferent signals (see Fig. 6). This feature can be described by associative memory functions of the nervous system. Some conditioned reflexes may cause “inappropriate” or “pathological reactions.” Consequently, a *circulus vitiosus* of pain sensation and muscle and/or vessel contraction is generated when pain becomes chronic (pain spiral). The key feature is the dedicated engram responsible for the pathological (painful) reaction. Pain memory can be explained by the concept of a holographic memory model that has been published by several authors.

A bio-cybernetic concept associatively linking sensory input and motor output to a reflex bow was published by Wess and Röder^[34]^.

**Figure 7.**
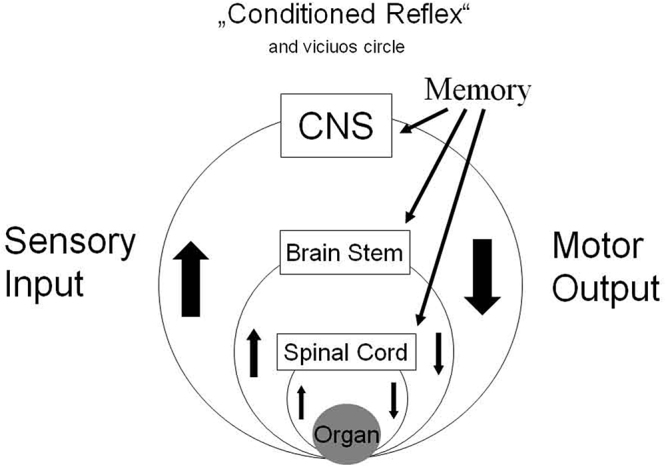
Schematic view of establishing conditioned reflexes by associative memory patterns on different levels of the CNS.

If we consider conditioned reflexes as response of a dedicated motor output linked to a specific sensory input, we can conclude that the conditioning procedure left modifications in the CNS and built a memory trace (see Fig. 7). Whenever the accordant sensory input occurs, a linked motor action is initiated (Pavlov’s dog).

This type of linkage functions as an “associative memory.”

The concept of synaptic memory is based on the following findings and assumptions:

- Metabolism of intensely stimulated synapses is enhanced (long-term potentiation)
- Repeatedly stimulated synapses facilitate transmission of signals.
- Neural interference patterns are permanently stored in the excitability (threshold) patterns of large numbers of cells and synapses.

We hypothesize that for many chronic diseases, pathological muscle spasm is attributed to permanent neural activity that sends frequent motor signals to the affected muscle (circulus vitiosus), causing permanent muscle contraction and spastics, which, in turn, reduces blood flow and metabolism. The therapeutic goal is to release the *circulus vitiosus* of permanent pain sensation and contracting motor signals.

According to this hypothesis, the key to effective shock wave treatment is to target a reorganization of the pathologic memory traces in the CNS.

Reorganization or distinction of pathologic memory traces by **hyperstimulation** is considered the mechanism of pain relief. Strong repeated nerve stimuli activate the synapses involved in releasing stored transmitter-substances into the synaptic gap.

Extensive stimulation leads to an exhaustive release of transmitter-substances to the point of diminished or inhibited response of the connected nerve cells. The link between sensory input and motor output of the pathological reflex (circulus vitiosus) is interrupted, and painful effects (muscle or vessel tonus) are released. (For further details, see^[33]^).

In a typical shock wave treatment (shoulder pain, heel spur, tennis elbow, etc.), the point of maximum pain sensation (directed by the patient’s response) is stimulated until an analgesic effect^[35]^ a numb feeling occurs. Through dialogue with the patient, the next painful point was identified and treated until numbness was felt again. At least for the recovery time of the synaptic gap, until the synaptic vesicles are re-charged with transmitter substances, the vicious circle is interrupted and pain sensation is reduced. Repeated treatment sessions strengthen the modified pain memory (learning process) and provide persistent pain relief.

The whole hypothesis is based on the fact that shock waves generate pulsed mechanical forces appropriate to open cell membranes by the mechanism of **mechano-sensory-transduction**. Unlike continuous wave (CW) ultrasound, shock waves feature significant momentum that generates forces by reflection (momentum transfer) at tissue interfaces. These forces act on the inertial mass of the affected tissue and stretch and squeeze the cell membranes within milliseconds. Millisecond pulses match favorably with the excitation time of nerve cells to generate action potentials, which are sent via nerve fibers to the CNS. In comparison with the microsecond forces generated by CW ultrasound, these forces are too fast (see above) to mechanically open cell membranes.

Thus, stimulating peripheral nerve cells is mandatory for modifying the centralized pain memory in the CNS.

### Transcranial neuro stimulation

Because stimulation of peripheral nerves has an impact on selected processes in the brain, one can hypothesize that direct stimulation of the CNS may activate and re-activate nerve cells in the brain. For example, Alzheimer’s disease is associated with the loss of neural cell functions. The etiological factors for Alzheimer`s disease worldwide are a major challenge in medical research and are still under debate.

TPS is a new therapeutic approach for exerting stimulating forces on nerve cells with alleviated functions.

The extracorporeally generated shock wave pulses are stimulated via the mechanism of momentum transfer (see above) nerve cells in the brain, re-activating and increasing the metabolism of the affected synapses. Frequent repetition of stimulating pulses may lead to enhanced transmission of nerve signals through synapses and can be considered a learning effect.

**Figure 8.**
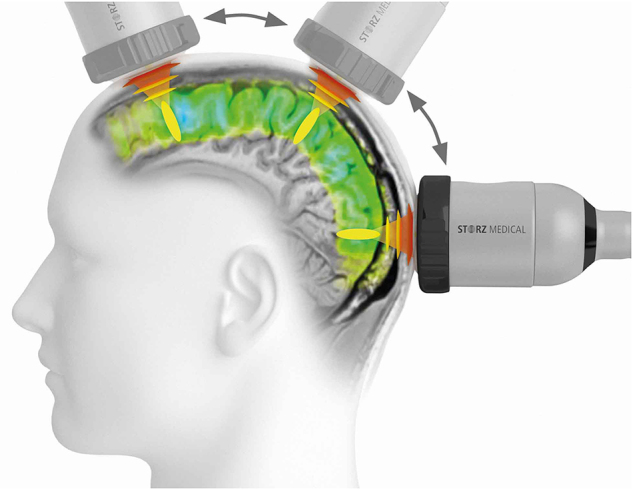
Schematic view of transcranial shock wave application.

The TPS uses pulse waves that are directly applied through the cranial bone to the brain (see Fig. 8). The effect of TPS was reported in various publications by Beisteiner^[24]^.

The authors used pulse waves, which are low-energy shock waves that are identical to the shock waves mentioned above. In a broader sense, they can be classified as ultrasound.

TPS by mechano-sensory transduction offers a novel approach to improve Alzheimer’s complaints based on the direct stimulation of nerve cells in the brain.

According to the calculations and measurements described above, the momentum of a shock wave is the basic mechanism of the features, fragmentation, and stimulation processes. The momentum of the shock wave is partly transferred from the shock wave to mass m of the affected body by acceleration to velocity *v* (*P = mv*) (see example). Movements of the affected inertial mass are in the range of several cm/s and are sufficiently slow to act on cell membranes to open ion channels and react with chemical or electrical responses. Physiological processes are triggered by millisecond shock wave stimuli, whereas microsecond signals, for example, by ultrasound waves, do not respond significantly. Based on Newton’s laws of movement, momentum transfer generates forces at acoustic interfaces that are matched by twice the reflection coefficient *R* (force *F* = 2*RP*/∆*t*). The same shock wave acts powerful on strong reflectors (*R* ≈ 1) and soft on weak reflectors (*R*«1)

This beneficial feature of shock waves lays the groundwork for numerous established shock wave therapies and legitimates the development of a new hypothesis for the neural mechanism of shock wave treatment in Alzheimer’s disease.

## Discussion

With the new concept of momentum transfer, we gain a detailed understanding of the physical mechanism of shock waves in medicine. To the best of our knowledge, this is the first study to define and calculate the forces responsible for the fragmentation of body stones and, simultaneously, for mechanotransduction, which is widely considered as the biological mechanism for many healing effects. Momentum transfer shifts part of the momentum of the incident shock wave to the momentum of the affected tissue. This means that forces generated at acoustic interfaces move, squeeze, and strain the tissue, which is the precondition for mechanotransduction. Momentum transfer implies that the shock waves feature momentum. This was not recognized until recently^[29]^.

Based on this mechanism, shock waves possess a favorable feature: their forces are generated only at the interfaces of acoustically different tissues. In soft tissues with small impedance differences (skin, muscle, fat, etc.), no, or only small forces are generated. This means that a shock wave can pass layers of soft tissues without significant reflection, energy loss, or tissue lesions unless it hits solid (or gaseous) body structures with higher or significantly lower impedances, such as stones or lunges. These interfaces are characterized by a high reflection factor, R. Therefore, the fragmentation effect predominately occurs at the stone surfaces. At gaseous interfaces (lung and bowls), high reflection forces may rupture the lung or bowl tissue. Gas-containing organs should be kept clear from the shock wave path. The reflection coefficient R defines the ratio of reflection and is adjacent to momentum, which is the most significant parameter for force generation.

In the case of non-destructive application of shock waves on soft tissues and bones, the shock wave-generated forces are due to the smaller reflection coefficient being weaker but strong enough to stimulate mechanotransduction processes. They stimulate the release of various bio-chemicals, such as endothelial nitric oxide synthase (eNOS), bone morphogenetic protein (BMP), vascular endothelial growth factor (VEGF), and substance P.

An additional promising effect of shock waves is the possibility to modify control functions in in the brain (CNS) by stimulation of peripheral nerves (PNS).

In most shock wave applications, the stimulation of nerve cells occurs from subtle pressure touch to a significant pain sensation. Afferent nerve signals reach higher levels in the CNS and initiate processes that adequately respond to peripheral signals. The response spectrum can be a manifold. Motor control functions, such as muscle contraction/relaxation and vasocontraction/dilatation, occur when shock waves are applied to living tissues.

With non-invasive shock wave application, it is possible to modify reflex patterns in the brain, as in the case of chronic pain release by hyper stimulation. This method may open a new field of “sensory motoric” diseases to be treated with shock waves.

In the case of Alzheimer’s disease, shock waves are hypothesized to stimulate nerve cells with alleviated functions and activate the metabolism of the shock wave-stimulated synapses.

This non-chemical and non-invasive treatment approach promises significant progress in Alzheimer therapy.

## Conclusion

We investigated the physical basis of shock wave therapy in medicine. The main question is how shock waves generate forces in living tissues and how they interact with different types of tissues. Momentum transfer provides answers on questions such as “why is the effect of equal shock waves different in different tissues,” “why shock waves can break stones without significant lesions in the surrounding tissue,” and “why shock waves can stimulate nerve cells better than similar ultrasound.”

Momentum transfer by reflection of shock waves at acoustic interfaces of biological tissue generates adequate forces for fragmentation of stones and the mechanism of mechano-transduction and mechano-sensory transduction, which may be the basis for healing various diseases in response to shock wave therapies. As shock waves stimulate nerve cells, the CNS is involved in the healing process. Shock wave therapy is lifted from a purely mechanical level to a higher level of complemented neuronal control functions.

Mechano-sensory-transduction explains how shock waves stimulate peripheral nerves, which activate and modify memory patterns in the brain. Hyperstimulation extinguishes “false” or “pathological” patterns which can be replaced by “re-learned, healthy” patterns.

Non-invasive and non-chemical shock wave therapy TPS is a new alternative and promising treatment modality for patients with Alzheimer’s disease, without chemical side effects.

## Limitations

This study is limited because only the physical part of the momentum transfer is experimentally proven. Medical aspects are hypothetical and difficult to be verified.

However, to date, no sound theory of the working mechanism of shockwaves in medicine has been established. With the help of the theory of momentum transfer, we gained a deeper insight into the healing processes that can test concepts in the medical world. Further investigations are required.

## Data Availability

This article is available upon reasonable request.
